# Patient advocates respond to ‘Utilizing Patient Advocates…’ by Feeney et al

**DOI:** 10.1111/hex.13087

**Published:** 2020-06-05

**Authors:** Sara Riggare, Benjamin Stecher, Jon Stamford

**Affiliations:** ^1^ Uppsala University Uppsala Sweden; ^2^ Independent Parkinson’s Patient Advocate Sweden; ^3^ Independent Parkinson’s Patient Advocate Canada; ^4^ Independent Parkinson’s Patient Advocate UK

We read with interest the recent article by Feeney and colleagues on ‘utilizing (sic) patient advocates in Parkinson's disease’.^1^ We acknowledge that sound methods for patient engagement need to be developed and evaluated. This can be especially relevant for Parkinson's disease (PD), a field we three know well from many years’ experience as active patient advocates living with PD. However, these methods need to be based on relevant premises. The article focuses on drug development and, for that reason alone, falls short of reflecting the full experience of PD upon which patient engagement needs to be constructed. Patient engagement, as defined by patients, reflects a wider scope of thought and experience than when defined by people who are not themselves patients.^2^ Before we start developing methods, we need a cultural and ideological shift across the field towards an acceptance that involving patients in therapeutic development is self‐evident.

The face of patient advocacy has changed. Patients today are no longer passive recipients of care^3^, ^4^ but, instead, take active roles in the management of symptoms^5^ and administration of therapies and treatments,^6^ developing new tools, delivery methods and interventions,^7^ even pushing the boundaries in research.^8^

Parkinson's disease also has its share of active patients, vocal campaigners for patient engagement. For example, Tom Isaacs who co‐founded a charity that ‘has not only funded ground‐breaking research and trials, but has fundamentally changed how we think about trying to better translate therapies to patients with Parkinson's’.^9^ In our roles as patient advocates in PD, we have contributed to expanding the knowledge, both in generating hypotheses^10^ and in real‐world applications.^11^, ^12^

Whilst we found the work described by Feeney et al^1^ interesting, we nonetheless have some serious reservations and feel that the paper reflects some long‐standing biases in the medical field that have held patient engagement back.

Firstly, the article discusses patient engagement as a commodity or treatment rather than a philosophical approach. Patient engagement is not an intervention to be compartmentalized, constrained and evaluated. Rather it is a prerequisite for a people‐centred health‐care system^13^ and a crucial element in the culture change needed.

As best we can see, no PD patients are authors on the paper, only charity officers and a neurologist. Nor are any patients mentioned in the acknowledgements. Whilst we can appreciate the application of academic rigour to the investigation, it is nonetheless absurd that patient engagement should be defined by charity officers or academics to meet their needs rather than evolving organically from the needs of the patient community. It is an example of the kind of cultural patrimony in medicine that patient engagement has sought to abolish.

Engaged patients are as essential to proper therapeutic development in long‐term chronic conditions as biochemists; their integration into the process is self‐evident. Whilst better metrics of engagement is a small step in the right direction, what everyone involved really needs to embrace is a cultural and ideological shift. Patient engagement in therapeutic development does not need justification, what we need are real‐world models of engagement.

To that point, we would like to address the case study highlighted in the article presumably as a good example of engagement. To allude to bringing in six patients, without any indication as to their level of expertise or actual input, for one day as a model is a telling sign of the disrespect such engagement strategies have for the role of the patient. Moreover, the primary measure of success seems to have been little more than the publication of two papers.

Imagine, if you will, the uproar that would have ensued if a maker of women's products, staffed only by men, who for decades had all failed to make anything that significantly improved the quality of life for women, was lauded for trying to remedy their failures by bringing together six women for one day and then writing a journal article or two about it.

Whilst buoyed that engagement strategies are at least being given more thought, we are not content with half‐measures in the right direction. We seek a cultural and ideological shift away from coddled attempts to ‘utilize patient advocates’ for short periods of time and towards efforts that seek to engage patient expertise in the same manner that one would engage any other expertise.

Our advice for those looking for ways to involve patients, whether in academia or industry (in both of which, mind you, patients need to be represented at all levels), is to just do it. Hire patients in your practice, make them part of your teams, and look to them early and often for consultation and advice. You will find that many, and they are not hard to find, are eager to engage with you on a professional level.

## CONFLICT OF INTEREST

All authors declare that they have no conflicts of interest.

## Data Availability

Data sharing is not applicable to this article as no new data were created or analysed in this study.
